# Investigating Direct Links between Depression, Emotional Control, and Physical Punishment with Adolescent Drive for Thinness and Bulimic Behaviors, Including Possible Moderation by the Serotonin Transporter *5-HTTLPR* Polymorphism

**DOI:** 10.3389/fpsyg.2017.01361

**Published:** 2017-08-09

**Authors:** Vanja Rozenblat, Joanne Ryan, Eleanor H. Wertheim, Ross King, Craig A. Olsson, Isabel Krug

**Affiliations:** ^1^School of Psychological Sciences, Faculty of Medicine, Dentistry and Health Sciences, The University of Melbourne, Parkville VIC, Australia; ^2^Murdoch Childrens Research Institute, Royal Children’s Hospital Melbourne, Parkville VIC, Australia; ^3^School of Psychology and Public Health, Faculty of Health, La Trobe University, Melbourne VIC, Australia; ^4^School of Psychology, Faculty of Health, Centre for Social and Early Emotional Development, Deakin University, Geelong VIC, Australia; ^5^Department of Paediatrics, The Royal Children’s Hospital Melbourne, Faculty of Medicine, Dentistry and Health Sciences, The University of Melbourne, Parkville VIC, Australia

**Keywords:** *5-HTTLPR*, gene–environment interactions, disordered eating, parental physical punishment, depression, emotional control

## Abstract

**Objectives:** To examine the relationship between psychological and social factors (depression, emotional control, sexual abuse, and parental physical punishment) and adolescent drive for Thinness and Bulimic behaviors in a large community sample, and to investigate possible genetic moderation.

**Method:** Data were drawn from the Australian Temperament Project (ATP), a population-based cohort study that has followed a representative sample of 2443 participants from infancy to adulthood across 16 waves since 1983. A subsample of 650 participants (50.2% female) of Caucasian descent who provided DNA were genotyped for a serotonin transporter promoter polymorphism (*5-HTTLPR*). Adolescent disordered eating attitudes and behaviors were assessed using the Bulimia and Drive for Thinness scales of the Eating Disorder Inventory-2 (15–16 years). Depression and emotional control were examined at the same age using the Short Mood and Feelings Questionnaire, and an ATP-devised measure of emotional control. History of sexual abuse and physical punishment were assessed retrospectively (23–24 years) in a subsample of 467 of those providing DNA.

**Results:** EDI-2 scores were associated with depression, emotional control, and retrospectively reported parental physical punishment. Although there was statistically significant moderation of the relationship between parental physical punishment and bulimic behaviors by *5-HTTLPR* (*p* = 0.0048), genotypes in this subsample were not in Hardy–Weinberg Equilibrium. No other G×E interactions were significant. **Conclusion:** Findings from this study affirm the central importance of psychosocial processes in disordered eating patterns in adolescence. Evidence of moderation by *5-HTTLPR* was not conclusive; however, genetic moderation observed in a subsample not in Hardy–Weinberg Equilibrium warrants further investigation.

## Introduction

Eating disorders (EDs) are believed to have a substantial heritable component (Bulik et al., 2016), with estimates from twin studies ranging from 40 to 60% (Yilmaz et al., 2015). Thus far, research examining molecular genetic mechanisms that may increase risk for eating pathology has largely investigated whether certain genetic polymorphisms (e.g., serotonin transporter linked polymorphism, *5-HTTLPR*) are found in different frequency in those with a clinical ED compared to controls. These studies have largely produced inconsistent findings (Calati et al., 2011; Solmi et al., 2016), supporting the notion that genetic risk operates in a manner more complex than simple association. One line of research receiving increasing attention is the possibility that certain polymorphisms may induce differential risk, depending upon exposure to certain environmental factors, via gene by environment (G×E) interaction.

Studies examining whether G×E interactions play a role in ED etiology have largely focussed on *5-HTTLPR*, with the short (s) allele associated with lower serotonin transcription activity compared to the long (l) allele (Heils et al., 1996). Serotonin plays a role in mood regulation, appetite, and weight (Blundell, 1984; Leibowitz and Alexander, 1998; Kalra et al., 1999; Ruhé et al., 2007), all known to be involved in eating pathology. Serotonin is also involved in the stress-response system (Gotlib et al., 2008; van Eekelen et al., 2012), and as *5-HTTLPR* is a functional polymorphism it may conceivably play a role in EDs, directly or indirectly through interaction with other environmental stressors. However, in other fields of psychiatry, the role of *5-HTTLPR* in moderating the effects of environmental stressors remains controversial; for example, in depression, where independently conducted meta-analysis continue to contradict one another (cf. Munafò et al., 2009; Risch et al., 2009; Karg et al., 2011). Lack of consensus stems from a range of methodological limitations, such as insufficient sample size, inappropriate statistical techniques and multiple testing, as well as substantial publication bias favoring significant G×E findings (Duncan and Keller, 2011; Duncan et al., 2014; Dick et al., 2015; de Vries et al., 2016).

To date, seven publications have investigated the role of G×E interactions in the ED field involving *5-HTTLPR* (Rozenblat et al., 2017). Systematic review and meta-analysis of these studies suggested that *5-HTTLPR* may moderate the risk relationship between experiencing both sexual and physical abuse and bulimic symptomatology (combined *N* = 1,096), and traumatic life events and ED symptomatology (*N* = 909). This was not the case, however, for risk relationships between depressive and bulimic symptomatology (*N* = 1254) or impulsivity and disordered eating (*N* = 1122). Findings from this review suggest that risk associated with *5-HTTLPR* may be intensified under increasingly severe social stress, but not psychological distress.

However, findings from this work were based on a combined sample that was derived by summing across small highly heterogeneous samples [e.g., two community samples, *N* = 369, Akkermann et al., 2012; *N* = 623, van Strien et al., 2010; a clinical sample, *N* = 89, Richardson et al., 2008; and a discordant sister-pair sample, *N* = 168 from European cross-institutional data set used in Karwautz et al. (2011)]. Testing interactions in one large, homogenous sample would be preferable (Cochran, 1954). This, for example, may explain why *5-HTTLPR* was found to moderate the effects of depression on eating outcomes in two of the original studies (van Strien et al., 2010; Mata and Gotlib, 2011) but not in the combined data analysis (Rozenblat et al., 2017). Furthermore, the lack of significant interaction between impulsivity and *5-HTTLPR* in the combined-sample may be partly due to the analysis of impulsivity as an overall construct, rather than separately testing the particular facets of impulsivity that have previously been associated with EDs, such as negative urgency (Racine et al., 2009, 2013). Negative urgency refers to the tendency to act rashly or feel strong impulses when experiencing negative affect (Whiteside and Lynam, 2001), and, along with the broader ability to regulate one’s emotions, has wide empirical support for a role in EDs, particularly bulimia nervosa symptomatology (Fischer et al., 2003; Claes et al., 2005), with some evidence linking emotional regulation and *5-HTTLPR* function (Hariri and Holmes, 2006). From a theoretical perspective, lowered emotional control may lead to greater eating pathology, as individuals may attempt to control their emotional states via altered food intake (e.g., binge eating or restricted intake; Haynos and Fruzzetti, 2011; Pearson et al., 2015). Meanwhile, the other psychological factor that has been analyzed in a G×E framework, depressed mood, is believed to precipitate bulimic behaviors under a number of key ED models (e.g., Dual Pathway Model; Stice, 2001) with support from longitudinal investigations of high-risk samples (Stice et al., 2017), although there is evidence suggesting depressed mood may also arise as a consequence of eating pathology (Puccio et al., 2016).

While many prior studies investigating G×E interactions have focussed on patients with clinical EDs (Rozenblat et al., 2017), analysis of disordered eating in community samples is of equal, if not greater importance. Developing a better understanding of the correlates and risk factors for pre-clinical eating pathology, which may later develop into a ‘full blown’ ED (Herpertz-Dahlmann et al., 2013), can help promote prevention at the earliest possible opportunity to reduce ED incidence (Stice et al., 2007). From a practical perspective, this also allows for the collection of far larger samples compared to studies using case-control designs, which is a key consideration in genetic association research (Duncan and Keller, 2011).

To further test preliminary findings from Rozenblat et al. (2017) in a homogenous sample, the present study used data from 650 participants who provided DNA in the Australian Temperament Project (ATP), a population based cohort study that has followed a representative sample of around 2000 participants from infancy to adulthood since 1983. The first aim was to assess the direct effects of depressed mood, emotional control, sexual abuse, and parental physical punishment on adolescent drive for thinness and bulimic behaviors. The second aim was to examine the extent to which the relationships between these factors and eating pathology were moderated by *5-HTTLPR*. Results of this study constitute an important step toward accumulating evidence regarding whether genetic factors may moderate the influence of psychological and environmental risk factors on EDs.

## Materials and Methods

### Participants

Australian Temperament Project participants were initially recruited in infancy (4–8 months) in Victoria in 1983, using a stratified random sampling framework, via maternal and child health centers in urban and rural locations. The first survey included 2,443 infants (48.0% female), with 16 surveys completed to date. The present study involved a sub-set of 650 participants (50.2% female) who had completed the 11th survey at age 15–16 years, providing information on drive for thinness and bulimic behaviors, depressive symptoms and emotional control, and who also provided a saliva sample for genotyping at this time (*N* = 567), or in their early 30s (*N* = 83, in 2015 as part of a separate sub-study). Of the 650 participants, 467 participants also provided retrospective information regarding sexual abuse and parental physical punishment in the 14th survey at age 23–24, and were included in the respective analyses. To avoid issues related to genetic heterogeneity, 23 participants who self-identified as non-Caucasian were excluded prior to forming the sample. The final analysable sample had a higher proportion of participants from the highest SES quartile than the original sample (for full socio-demographic information, see **Table 1**). Due to missing data, the final sample comprised 643 participants for the depression analyses and 649 for the emotional control analyses. Parents and adolescents provided written informed consent for each survey wave and for the collection of saliva samples. The data collection was approved by the Australian Institute of Family Studies Ethics Review and carried out in accordance with the latest version of the Declaration of Helsinki.

**Table 1:** Sociodemographic details of participants included in the present sample.

	Full sample			Females		Males	
	*N*	*N*	%	*N*	%	*N*	%
Sex	650						
Males		312	49.8				
Females		338	50.2				
SES Quartile	632						
Highest		231	36.6	116	36.6	115	36.5
Medium-High		197	31.2	103	32.5	94	29.8
Medium-Low		129	20.4	59	18.6	70	22.2
Lowest		75	11.9	39	12.3	36	11.4
Parent marital status	645						
Married/*De facto*		538	83.4	264	81.5	274	85.4
Separated/Divorced		73	11.3	42	13.0	31	9.7
Single/Widowed		22	3.4	10	3.1	12	3.7
Remarried		12	1.8	8	2.5	4	1.2
Father’s occupation	612						
Professional		172	28.1	85	29.1	87	29.6
Managerial		126	20.6	61	20.9	65	22.1
Semi-skilled		200	32.7	105	36.0	95	32.3
Unemployed/Pensioner/House duties		114	18.6	41	14.0	47	16.0
Mother’s occupation	637						
Professional		175	27.3	80	25.4	95	30.4
Managerial		54	8.5	31	9.8	23	7.3
Semi-skilled		193	30.3	104	33.0	89	28.4
Unemployed/Pensioner/House duties		215	33.8	100	31.7	106	33.9
Father’s education	594						
Tertiary		176	29.6	86	28.7	90	30.6
Diploma/Apprenticeship		103	17.3	49	16.3	54	18.4
Year 11/12		167	28.1	87	29.0	80	27.2
Year 10 or less		148	24.9	78	26.0	70	23.8
Mother’s education	633						
Tertiary		133	21.0	66	21.0	67	21.1
Diploma/Apprenticeship		102	16.1	55	17.5	47	14.8
Year 11/12		227	35.9	116	36.8	111	34.9
Year 10 or less		171	27.0	78	24.8	93	29.2

### Measures

#### Disordered Eating

Drive for thinness and bulimic behaviors were assessed at age 15–16 via the Eating Disorder Inventory-2 (EDI-2; Garner, 1991) Drive for Thinness and Bulimia scales. The Drive for Thinness scale consists of 7-items measuring participants’ desire to lose weight or fear of weight gain (e.g., “I am preoccupied with the desire to be thinner”). Internal consistency in the current sample was α = 0.92. The Bulimia scale consists of 7 items measuring bulimic behaviors, including binging and purging (e.g., “I stuff myself with food”), with Cronbach’s α = 0.74 in the current sample. For details of scoring and some minor modifications made for an Australian context, refer to Krug et al. (2016).

#### Psychological Stress Exposures

Depression was assessed at the same time-point as disordered eating via the Short Mood and Feelings Questionnaire (SMFQ) (Angold et al., 1995), a 13-item subscale derived from the original 33-item questionnaire. The SMFQ is intended as a screening measure for children and adolescents that queries depressive symptoms according to DSM-III criteria (American Psychiatric Association [APA], 1980; e.g., “I feel miserable or unhappy”), with responses provided on 3-point scale (*rarely/never, sometimes, often/always*). Participants with missing data on five or more items were excluded from analyses (α = 0.83 in current sample).

Emotional control measured participants’ capacity to control their emotions and was also assessed at age 15–16 using an ATP-devised measure consisting of 10-items (e.g., “I am able to keep my feelings under control” and “I am able to calm down if I am feeling nervous”) rated on a 6-point scale from *never* to *always*. This measure has been previously used in studies examining internalizing problems (Toumbourou et al., 2011), with α = 0.70 in the present sample.

#### Sexual and Physical Stress Exposures (Retrospective)

A number of retrospective indicators were used at age 23–24 to assess sexual abuse and parental physical punishment during childhood and adolescence. Sexual abuse was based on a ‘yes’ response to the questions: “You had a sexual experience with a person who was not a family member prior to 16” and a follow up ‘no’ response to the question “Was this consensual?”, or, a ‘yes’ response to the question “A family member did, or tried to do, sexual things to you.”

Mild-to-moderate parental physical punishment was based on a ‘yes’ response to the question “Your parent/s used harsh physical treatment (e.g., smacking, hitting) to discipline you,” and severe parental physical punishment was based on an additional ‘yes’ response to a follow up question, “Did you ever suffer effects that lasted to the next day or longer (e.g., bruising, marking, pain, soreness)?”, creating two distinct severity categories.

#### *5-HTTLPR* Genotyping (Moderation Variable)

Following the 11th survey, DNA for 567 participants was isolated using Qiagen QIAamp kits from buccal epithelial cells via cotton swabs, with further details described in Jorm et al. (2000). Saliva samples for an additional 83 participants were collected following the 16th survey in 2015 using Oragene saliva pots or tubes and analyzed at the Australian Genomics Research Facility (AGRF), Adelaide, SA, Australia. Genotype frequencies were similar in the original and 2015 samples. For all samples, *5-HTTLPR* genotype was coded as per the di-allelic model into s-present (s/s or s/l genotype) or s-absent (l/l genotype) groups, as the s-allele is believed to operate in a genetically dominant manner (Lesch et al., 1996).

#### Potential Confounding Factors

Age, height, and weight were self-reported at age 15–16, with the latter two figures used to calculate participant BMI. SES status was measured according to maternal and paternal education and occupation as reported by parents in the first survey in 1983.

### Data Analysis

The main and interaction effects of *5-HTTLPR* and the two psychological stress exposures (depression and emotional control), as well as the three social stress exposures (sexual abuse, mild-to-moderate parental punishment, and severe parental physical punishment), were assessed using separate linear regression models. Outcome variables were Drive for Thinness and Bulimia scores. G×E models were adjusted for sex and BMI, as per Keller (2014), by including all the covariate × gene and covariate × environment interaction terms in the regression models. Prior to analyses, missing data (23.5%) for the BMI variable were imputed using multiple imputation in IBM SPSS Version 21, with no systematic patterns of missingness observed. A total of 10 tests were conducted with *p*-values adjusted accordingly (adjusted *p*-value = 0.05/10, corrected *p* = 0.005), to correct for multiple-testing, a frequent limitation of genetic association studies (Munafò and Flint, 2009). Standardized effect sizes are reported.

## Results

*5-HTTLPR* genotype distribution (l/l = 197, s/l = 341, and s/s = 112) for the overall sample met the Hardy–Weinberg Equilibrium, χ^2^ = 2.96, *df* = 1, *p* > 0.05. *5-HTTLPR* genotype distribution was not in Hardy–Weinberg Equilibrium for the subsample providing retrospective reports of sexual and physical stress (l/l = 139, s/l = 255, and s/s = 73; χ^2^ = 6.11, *df* = 1, *p* = 0.014). Further descriptive statistics are presented in **Table 2**. Across all regression models, female sex predicted Drive for Thinness and Bulimia scores, while BMI predicted Drive for Thinness (all *p* < 0.001). Tables pertaining to each regression model discussed below are contained in the Supplementary Materials.

**Table 2:** Descriptive statistics for mean values of continuous predictor and outcome variables in the overall sample (*N* = 650), in females (*N* = 326), and in males (*N* = 324).

	Mean (*SD*)		
Variable	Full sample	Females	Males
Age (years)	15.72 (0.16)	15.74 (0.14)	15.72 (0.17)
EDI-2 Bulimia	1.78 (0.65)	1.94 (0.73)	1.62 (0.53)
EDI-2 drive for thinness	2.23 (1.15)	2.81 (1.22)	1.64 (0.70)
Emotional control	3.74 (0.63)	3.62 (0.64)	3.85 (0.59)
Depression	0.49 (0.34)	0.59 (0.36)	0.39 (0.28)
BMI	21.27 (3.28)	21.20 (3.10)	21.34 (3.44)

### Depression

There was a significant positive association between depressive symptoms and Drive for Thinness (β = 0.24, *p* < 0.001), as well as Bulimia scores (β = 0.41, *p* < 0.001); however, there was no evidence of genetic moderation by *5-HTTLPR*. There was a significant interaction between depression and sex, with depression associated with greater Drive for Thinness (β = 0.46, *p* = 0.001), and to a lesser extent, Bulimia (β = 0.36, *p* = 0.018), for females only. There were no other significant effects.

### Emotional Control

Lower emotional control was significantly associated with greater Drive for Thinness (β = -0.22, *p* < 0.001) and Bulimia (β = -0.29, *p* < 0.001) scores; however, there was no evidence of genetic moderation by *5-HTTLPR*. There was a significant interaction between sex and emotional control, with lower emotional control associated with greater Drive for Thinness (β = -0.45, *p* = 0.001) and Bulimia (β = -0.67, *p* < 0.001) for females to a greater extent than for males. However, amongst those with the highest levels of emotional control, females displayed lower levels of Bulimia than did males.

### Sexual Abuse and Parental Physical Punishment

Of the 467 participants (59.1% female) who provided data on sexual abuse and parental physical punishment in the 14th survey, 22 (4.7%) reported sexual abuse, 180 (38.5%) reported mild to moderate parental physical punishment, and 27 (5.8%) reported severed parental physical punishment. See **Table 3** for further descriptive statistics. Predictor and outcome variables for this sub-sample did not differ from the overall sample (*t*-tests all *p* > 0.05).

**Table 3:** Descriptive statistics for mean values of continuous predictor and outcome variables in the subsample (*N* = 467), in females (*N* = 199), and in males (*N* = 157).

	Mean (*SD*)		
Variable	Full sample	Females	Males
Age (years)	15.72 (0.15)	15.73 (0.15)	15.71 (0.14)
EDI-2 Bulimia	1.81 (0.67)	1.92 (0.68)	1.62 (0.51)
EDI-2 drive for thinness	2.37 (1.20)	2.79 (1.22)	1.69 (0.74)
Emotional control	3.74 (0.64)	3.64 (0.65)	3.88 (0.59)
Depression	0.51 (0.34)	0.58 (0.02)	0.40 (0.28)
BMI	21.19 (3.13)	21.17 (2.97)	21.21 (3.37)

There was a direct effect of severe parental physical punishment in predicting Bulimia (β = 0.14, *p* = 0.001), but not Drive for Thinness scores. There were no direct effects of sexual abuse or mild-to-moderate parental physical punishment on either disordered eating outcome, although there was an interaction between sexual abuse and sex (β = 0.28, *p* = 0.039), with males who reported experiencing sexual abuse tending to display lower Drive for Thinness than those who did not report sexual abuse. This pattern was not evident in females. However, this result did not withstand *p*-value adjustment for multiple testing.

**FIGURE 1:**
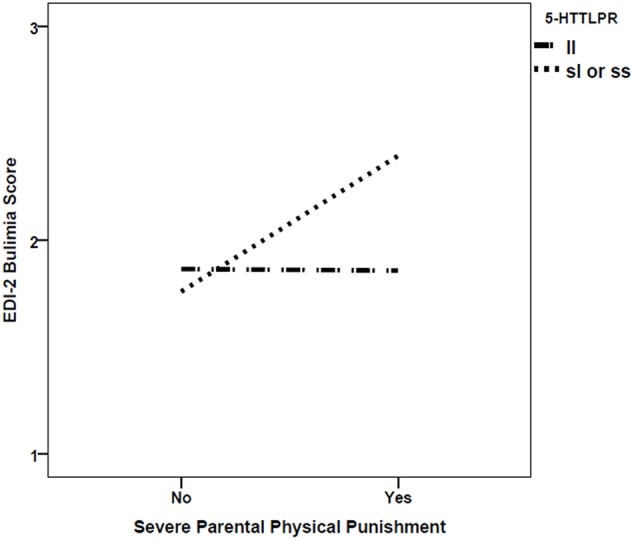
Interaction between *5-HTTLPR* short (s) allele and the experience of severe parental physical punishment in predicting EDI-2 Bulimia scores.

There was also statistically significant moderation of the relationship between severe parental punishment and bulimia scores by *5-HTTLPR*, with greater punishment related to higher Bulimia scores for those with the s-allele only (β = 0.22, *p* = 0.0048; see **Figure 1**). This finding remained after Bonferroni correction for multiple testing was applied. However, this result was based on a sample where genotypic frequencies were not in the Hardy–Weinberg Equilibrium, χ^2^ = 6.11, *df* = 1, *p* = 0.014. No other G×E interactions were significant.

## Discussion

This study affirmed the central relationship between depression, emotional control, and physical abuse and adolescent bulimic behaviors and attitudes regarding thinness. Conversely, *5-HTTLPR* did not directly predict any pattern of disordered eating, nor was there conclusive evidence that 5-*HTTLPR* moderated any risk factor for disordered eating. A statistically significant interaction between *5-HTTLPR* and retrospectively reported parental physical punishment was observed; however, genotypes in this subsample were not in Hardy–Weinberg Equilibrium so cautious interpretation and independent replication is needed.

Findings from this study support a key role for depression and compromised emotional control in adolescent drive for thinness and bulimic behaviors. The relationships differed by sex in most cases. Female sex predicted greater overall drive for thinness and bulimia, and sex differences were present in the relationships between depression, emotional control, and sexual abuse with disordered eating symptoms. Parental physical punishment was the only variable that showed no sex differences in its relationship to eating pathology. Results support the notion that correlates of and risk factors for disordered eating symptoms show substantial variation between males and females (Lewinsohn et al., 2002; Striegel-Moore et al., 2009). They also support past studies linking depression (Puccio et al., 2016) and emotional control (Svaldi et al., 2012) to ED symptomatology, and are aligned with theories proposing that individuals may engage in disordered eating in attempt to better regulate negative affect or other undesirable emotions (Stice, 2001; Haynos and Fruzzetti, 2011; Pearson et al., 2015). Overall, results highlight the importance of psychological factors and the role of the social environment in eating pathology.

The tentative suggestion that *5-HTTLPR* may moderate the relationship between severe, but not mild-to-moderate parental physical punishment and bulimic behaviors is reflected in findings from Rozenblat et al. (2017), which reported moderation of both sexual and physical abuse by *5-HTTLPR* in predicting bulimia-spectrum pathology, with strongest effects when both types of abuse were experienced. This suggests that further investigation in larger independent samples in Hardy–Weinberg Equilibrium (the expected allele distribution in a given population, deviation from which may compromise validity of results) may well support moderation of more extreme forms of adversity by *5-HTTLPR*. Further support for the idea that *5-HTTLPR* might moderate more severe forms of risk for disordered eating come from past studies in the ED field that found traumatic life events were associated with bulimic symptoms and disordered eating for individuals with the *5-HTTLPR* s-allele (Akkermann et al., 2012; Stoltenberg et al., 2012), and is reflected by the focus on traumatic life events and sexual abuse in the depression field (Nugent et al., 2011).

Lack of genetic moderation of depression or emotional control in predicting drive for thinness and bulimic tendencies is mostly consistent with previous findings (Rozenblat et al., 2017). These results align with the secondary data analysis in Rozenblat et al. (2017), and suggest that the null findings for depression and impulsiveness reported in the secondary data analysis were likely not due to sample heterogeneity or use of the broad impulsiveness variable as opposed to examining a personality construct that is more closely associated with eating pathology, such as emotional control. However, sample size limitations mean that the presence of small effects cannot be entirely ruled out and further investigation in larger samples remains important.

The lack of genetic moderation reported for depression does, however, contradict the significant G×E interactions between depression and *5-HTTLPR* identified in two past studies (van Strien et al., 2010; Mata and Gotlib, 2011); however, the sample of Mata and Gotlib (2011) (*N* = 50) was very low for investigation of genetic association (Duncan and Keller, 2011), while van Strien et al. (2010) examined emotional eating, which differed somewhat from the eating constructs measured in the present study. One possibility is that as psychological factors appear to be strong direct predictors of eating pathology, they may function as risk factors irrespective of *5-HTTLPR* genotype. In contrast, certain environmental factors may have a more tenuous association with ED symptoms and thus plausibly could increase risk primarily for individuals with a genetic susceptibility.

The absence of direct genetic association in this study also partly conflicts with previous findings (Calati et al., 2011; Chen et al., 2015). Direct genetic prediction of ED has been investigated in several past studies examining clinical populations with mixed results. Two meta-analyses identified a direct association between *5-HTTLPR* and eating pathology (Odds Ratio: 1.35, 95%CI: 1.07-1.71, Calati et al., 2011; Chen et al., 2015), although they examined almost entirely the same group of studies, while the largest and most recent meta-analysis on this topic reported no association (Solmi et al., 2016). Notably, these meta-analyses were limited by substantial heterogeneity, the inclusion of studies with very small sample sizes (*N* < 100), and omission of tests for publication bias. Publication bias is noted to be a major problem affecting studies of G×E interactions and contributing to false-positive findings (Duncan and Keller, 2011; de Vries et al., 2016), with such issues argued to most strongly affect studies with small sample sizes (Ioannidis et al., 2014).

### Strengths and Limitations

Strengths of the present study include use of a homogenous, high-quality data set, with measurement of drive for thinness and bulimic tendencies in a community sample. Results are therefore of key relevance to aiding prevention of the development of clinical-level eating pathology. A further strength was the fact that the present study constituted the second largest unified sample investigating G×E interactions in eating pathology, following Akkermann et al. (2011) (*N* = 767), with mean sample size of existing ED G×E studies *N* = 288 (Rozenblat et al., 2017). This study was powered to detect direct and interaction effects of moderate size, which would be of clinical significance if detected. In light of growing evidence that genetic effect sizes involved in psychiatric disorders are exceedingly small, even larger samples are desirable. Methodological issues include use of the di-allelic model of *5-HTTLPR*, with some evidence that the tri-allelic may better represent activity of this polymorphism (Wendland et al., 2006), as well as the use of self-report questionnaires to measure most constructs, with accounts of sexual abuse and parental physical punishment measured retrospectively. Accordingly, the measure of emotional control used in the present study does not have published psychometric properties, although it has been used in previous research (O’Connor et al., 2011; Toumbourou et al., 2011). Finally, *5-HTTLPR* is just one of numerous genetic factors that may be involved in the etiology of disordered eating.

### Implications and Future Directions

Findings from this study suggest that psychological and environmental variables remain central in eating pathology, while evidence for specific candidate genes continues to be tentative at best. Although a statistically significant genetic interaction effect was identified in this study, evidence remains inconclusive because the subsample on which it was based was not in Hardy–Weinberg Equilibrium. It is important to note, however, that the null results reported in this study sit in contrast to the substantial genetic contribution to most psychiatric outcomes estimated in twin study designs (Trace et al., 2013). This suggests that there is still much work to do in the area of eating pathology to adequately explain the variation reported in twin studies. Null findings from this study suggest a more complex picture of genetic determination, one that would benefit from a move to genome-wide approaches, with an emphasis on identifying polygenic effects that emerge from networks of genes, which may better reflect the genetic foundations of complex diseases. Future studies of candidate genes should prioritize increasing statistical power, which may be achieved via data sharing across consortiums of life-course studies. Studies such as the present investigation provide a valuable contribution that should form part of future meta-analytic investigations, and constitute an important step forward in progressing investigation of how psychosocial and genetic factors may be related to eating pathology.

## Author Contributions

VR was responsible for conducting all analyses and preparing all sections of the manuscript. IK, JR, EW, RK, and CO were involved in collecting data and revising the manuscript for important intellectual content. All authors contributed to and approved the final manuscript.

## Conflict of Interest Statement

The authors declare that the research was conducted in the absence of any commercial or financial relationships that could be construed as a potential conflict of interest.
