# Supplementary material for: Investigating Direct Links between Depression, Emotional Control, and Physical Punishment with Adolescent Drive for Thinness and Bulimic Behaviors, Including Possible Moderation by the Serotonin Transporter 5-HTTLPR Polymorphism
Source: Front Psychol. 2017 Aug 9;8:1361. doi: 10.3389/fpsyg.2017.01361 (PMC5552700; doi:10.3389/fpsyg.2017.01361)
Supplement: Supplementary file 1 [file Table_1.pdf]

**Supplementary Materials**

Tables 1 to 4 display the main effects of each environmental or psychological variable on EDI-2 Drive for Thinness and Bulimia scales, along with moderation by 5-HTTLPR. The regression models investigating 5-HTTLPR moderation include all gene x covariate and environment x covariate interaction terms, as specified by Keller (2014) as necessary to adequately control for the influence of these variables on the GxE interaction.

**Table 1**

Results of regression analyses investigating main and interaction effects of *5-HTTLPR* and depression on EDI-2 Drive for Thinness and Bulimia Scores

| Variable | B | Lower CI | Upper CI | Beta | *t*-value | Sig. |
|---|---|---|---|---|---|---|
| Main effects model: Drive for Thinness | | | | | | |
| Constant | -1.80 | -2.34 | -1.26 | | -6.59 | <.001 |
| Depression | .83 | .61 | 1.04 | .24 | 7.52 | <.001 |
| 5-HTTLPR | -.08 | -.23 | .07 | -.03 | -.101 | .313 |
| Gender | 1.01 | .86 | 1.16 | .44 | 13.60 | <.001 |
| BMI | .10 | .08 | .12 | .29 | 8.98 | <.001 |
| Interaction effects model: Drive for Thinness | | | | | | |
| Constant | -1.60 | -2.93 | -.89 | | -2.56 | .011 |
| Depression | .50 | -1.07 | 2.07 | .15 | .63 | .532 |
| 5-HTTLPR | -.11 | -1.31 | 1.09 | -.04 | -.18 | .858 |
| Depression x 5-HTTLPR | .03 | -.48 | .43 | -.01 | -.13 | .899 |
| Gender | .65 | .32 | .99 | .28 | 3.79 | <.001 |
| Depression x Gender | .79 | .34 | 1.23 | .46 | 3.48 | .001 |
| 5-HTTLPR x Gender | -.006 | -.33 | .31 | -.01 | -.04 | .968 |
| BMI | .12 | .07 | .18 | .34 | 4.28 | <.001 |
| Depression x BMI | -.04 | -.11 | .02 | -.29 | -1.29 | .199 |
| 5-HTTLPR x BMI | .002 | -.05 | .06 | .02 | .09 | .930 |
| Main effects model: Bulimia | | | | | | |
| Constant | 1.13 | .69 | 1.56 | | 5.31 | <.001 |

| | | | | | | |
|---|---|---|---|---|---|---|
| Depression | .79 | .66 | .93 | .41 | 11.25 | <.001 |
| 5-HTTLPR | -.01 | -.11 | .08 | -.01 | -.29 | .774 |
| Gender | .16 | .07 | .25 | .12 | 3.38 | .001 |
| BMI | .001 | -.02 | .02 | .01 | .15 | .880 |
| <u>Interaction effects model: Bulimia</u> | | | | | | |
| Constant | 1.23 | .37 | 2.10 | | 2.82 | .005 |
| Depression | .32 | -.79 | 1.42 | .16 | .57 | .573 |
| 5-HTTLPR | .08 | -.71 | .86 | .05 | .19 | .847 |
| Depression x 5-HTTLPR | .17 | -.13 | .46 | .09 | 1.10 | .272 |
| Gender | .05 | -.17 | .27 | .04 | .44 | .661 |
| Depression x Gender | .35 | .06 | .64 | .36 | 2.38 | .018 |
| 5-HTTLPR x Gender | -.08 | -.28 | .13 | -.09 | -.72 | .863 |
| BMI | .007 | -.03 | .05 | .04 | .37 | .710 |
| Depression x BMI | -.009 | -.06 | .04 | -.11 | -.38 | .706 |
| 5-HTTLPR x BMI | -.003 | -.04 | .03 | -.05 | -.17 | 863 |

*Note.* Confidence intervals (CIs) are set at 95%

**Table 2**

Results of regression analyses investigating main and interaction effects of *5-HTTLPR* and emotional control on EDI-2 Drive for Thinness and Bulimia Scores

| Variable | B | Lower CI | Upper CI | Beta | *t*-value | Sig. |
|---|---|---|---|---|---|---|
| <u>Main effects model : Drive for Thinness</u> | | | | | | |
| Constant | .11 | -.56 | .82 | | .31 | .759 |
| Emotional Control | -.41 | -.53 | -.30 | -.22 | -7.09 | <.001 |
| 5-HTTLPR | -.08 | -.23 | .08 | -.03 | -.973 | .331 |
| Gender | 1.09 | .94 | 1.23 | .47 | 14.66 | <.001 |
| BMI | .10 | .08 | .12 | .28 | 8.98 | <.001 |
| <u>Interaction effect model: Drive for Thinness</u> | | | | | | |
| Constant | -3.32 | -6.73 | .09 | | -1.91 | .057 |
| Emotional Control | .50 | -.38 | 1.37 | .23 | 1.12 | .264 |
| 5-HTTLPR | -.05 | -1.64 | 1.55 | .04 | -.06 | .953 |
| Emotional Control x 5-HTTLPR | .02 | -.24 | .27 | .23 | .12 | .903 |
| Gender | 2.61 | 1.74 | 3.49 | .91 | 5.84 | <.001 |
| Emotional Control x Gender | -.40 | -.63 | -.17 | -.45 | -3.43 | .001 |
| 5-HTTLPR x Gender | -.04 | -.36 | .28 | -.17 | -.25 | .801 |
| BMI | .15 | .009 | .29 | .31 | 2.10 | .037 |
| Emotional Control x BMI | -.01 | -.05 | .02 | -.21 | -.80 | .426 |
| 5-HTTLPR x BMI | .001 | -.05 | .05 | -.04 | -.05 | .959 |
| <u>Main effects model: Bulimia</u> | | | | | | |
| Constant | 2.44 | 1.96 | 2.93 | | 9.91 | <.001 |
| Emotional Control | -.30 | -.38 | -.22 | -.29 | -7.75 | <.001 |
| 5-HTTLPR | -.03 | -.13 | .07 | -.02 | -.53 | .597 |
| Gender | .25 | .16 | .35 | .19 | 5.17 | <.001 |
| BMI | .01 | -.01 | .02 | .02 | .61 | .540 |
| <u>Interaction effect model: Bulimia</u> | | | | | | |
| Constant | 1.09 | -1.10 | 3.29 | | .97 | .330 |
| Emotional Control | .02 | -.57 | .60 | .02 | .05 | .959 |
| 5-HTTLPR | .12 | -1.12 | 1.35 | .13 | .11 | .849 |

| | | | | | | |
|---|---|---|---|---|---|---|
| Emotional Control x 5-HTTLPR | .04 | -.11 | .20 | .28 | .41 | .682 |
| Gender | 1.30 | .72 | 1.89 | .79 | 4.36 | <.001 |
| Emotional Control x Gender | -.28 | -.427 | -.123 | -.67 | -3.54 | <.001 |
| 5-HTTLPR x Gender | -.04 | -.24 | .17 | -.20 | -.35 | .729 |
| BMI | -.005 | -.09 | .08 | .001 | -.11 | .911 |
| Emotional Control x BMI | .004 | -.02 | .03 | .13 | .34 | .737 |
| 5-HTTLPR x BMI | -.01 | -.06 | .04 | -.17 | -.46 | .648 |

*Note.* Confidence intervals (CIs) are set at 95%

**Table 3**

Results of regression analyses investigating main and interaction effects of *5-HTTLPR* and sexual abuse on EDI-2 Drive for Thinness and Bulimia Scores

| Variable | B | Lower CI | Upper CI | Beta | *t*-value | Sig. |
|---|---|---|---|---|---|---|
| <u>Main effects model: Drive for Thinness</u> | | | | | | |
| Constant | -1.72 | -2.67 | -.79 | | -3.77 | .001 |
| Sexual Abuse | .01 | -.43 | .45 | .002 | .05 | .957 |
| 5-HTTLPR | -.11 | -.32 | .09 | -.04 | -1.10 | .270 |
| Gender | 1.18 | .99 | 1.37 | .48 | 12.39 | <.001 |
| BMI | .11 | .07 | .15 | .29 | 5.48 | <.001 |
| <u>Interaction effect model: Drive for Thinness</u> | | | | | | |
| Constant | -1.69 | -3.15 | -.23 | | -2.28 | .024 |
| Sexual Abuse | .49 | -2.63 | 3.61 | .09 | .31 | .756 |
| 5-HTTLPR | -.17 | -1.82 | 1.48 | -.06 | -.20 | .843 |
| Sexual Abuse x 5-HTTLPR | .003 | -.88 | .88 | .001 | .006 | .995 |
| Gender | 1.12 | .77 | 1.47 | .46 | 6.22 | <.001 |
| Sexual Abuse x Gender | .98 | .05 | 1.92 | .28 | 2.06 | .039 |
| 5-HTTLPR x Gender | .04 | -.37 | .45 | .03 | .18 | .856 |
| BMI | .11 | .05 | .17 | .29 | 3.48 | .001 |
| Sexual Abuse x BMI | -.10 | -.25 | .06 | -.36 | -1.25 | .213 |
| 5-HTTLPR x BMI | -.001 | -.07 | .07 | -.004 | -.01 | .989 |
| <u>Main effects model: Bulimia</u> | | | | | | |
| Constant | 1.00 | .52 | 1.49 | | 4.05 | <.001 |
| Sexual Abuse | .14 | -.14 | .42 | .05 | .99 | .320 |
| 5-HTTLPR | -.06 | -.19 | .07 | -.04 | -.92 | .356 |
| Gender | .34 | .22 | .45 | .25 | 5.51 | <.001 |
| BMI | .02 | -.01 | .04 | .07 | 1.43 | .156 |
| <u>Interaction effect model: Bulimia</u> | | | | | | |
| Constant | .96 | -.001 | 1.91 | | 1.97 | .050 |
| Sexual Abuse | 1.28 | -.90 | 3.46 | .41 | 1.17 | .246 |
| 5-HTTLPR | -.10 | -1.27 | 1.08 | -.07 | -.16 | .870 |
| Sexual Abuse x 5-HTTLPR | .15 | -.43 | .72 | .03 | .50 | .621 |

| | | | | | | |
|---|---|---|---|---|---|---|
| Gender | .41 | .18 | .63 | .30 | 3.56 | <.001 |
| Sexual Abuse x Gender | .23 | -.37 | .83 | .12 | .75 | .456 |
| 5-HTTLPR x Gender | -.10 | -.37 | .16 | -.13 | .77 | .440 |
| BMI | .01 | -.03 | .05 | .06 | .57 | .568 |
| Sexual Abuse x BMI | -.08 | -.18 | .03 | -.51 | -1.45 | .153 |
| 5-HTTLPR x BMI | .009 | -.04 | .06 | .14 | .35 | .730 |

*Note.* Confidence intervals (CIs) are set at 95%

**Table 4**

Results of regression analyses investigating main and interaction effects of *5-HTTLPR* and mild-to-moderate and severe parental physical punishment on EDI-2 Drive for Thinness and Bulimia Scores

| Variable | B | Lower CI | Upper CI | Beta | *t*-value | Sig. |
|---|---|---|---|---|---|---|
| **Mild-Moderate Parental Physical Punishment** | | | | | | |
| Main effects model: Drive for Thinness | | | | | | |
| Constant | -1.70 | -2.64 | -.76 | | -3.71 | .001 |
| Moderate physical punishment | -.07 | -.26 | .12 | -.03 | -.77 | .441 |
| 5-HTTLPR | -.12 | -.32 | .08 | -.05 | -1.16 | .248 |
| Gender | 1.17 | .99 | 1.36 | .48 | 12.29 | <.001 |
| BMI | .11 | .07 | .15 | .29 | 5.56 | <.001 |
| Interaction effect model: Drive for Thinness | | | | | | |
| Constant | -1.73 | -3.37 | -.09 | | -2.09 | .039 |
| Moderate physical punishment | .09 | -1.58 | 1.76 | .04 | .11 | .911 |
| 5-HTTLPR | -.16 | -1.82 | 1.50 | -.06 | -.19 | .851 |
| Moderate physical punishment x 5-HTTLPR | -.07 | -.48 | .34 | -.03 | -.33 | .741 |
| Gender | 1.18 | .78 | 1.58 | .48 | 5.84 | <.001 |
| Moderate physical punishment x Gender | -.03 | -.42 | .36 | -.02 | -.15 | .883 |
| 5-HTTLPR x Gender | .01 | -.41 | .43 | .01 | .05 | .963 |
| BMI | .11 | .04 | .18 | .29 | 3.03 | .004 |
| Moderate physical punishment x BMI | -.004 | -.08 | .07 | -.03 | -.10 | .923 |
| 5-HTTLPR x BMI | .003 | -.07 | .07 | .02 | .07 | .945 |
| Main effects model: Bulimia | | | | | | |
| Constant | 1.01 | .52 | 1.50 | | 4.04 | <.001 |
| Moderate physical punishment | .04 | -.09 | .16 | .03 | .57 | .57 |
| 5-HTTLPR | -.06 | .34 | -.19 | -.04 | .07 | .335 |
| Gender | .34 | .22 | .46 | .25 | 5.52 | <.001 |
| BMI | .01 | -.01 | .04 | .07 | 1.36 | .178 |
| Interaction effect model: Bulimia | | | | | | |
| Constant | 1.21 | .18 | 2.24 | | 2.32 | .022 |
| Moderate physical punishment | -.33 | -1.33 | .68 | -.24 | -.64 | .525 |

| | | | | | | |
|---|---|---|---|---|---|---|
| 5-HTTLPR | -.11 | -1.28 | 1.05 | -.08 | -.19 | .848 |
| Moderate physical punishment x 5-HTTLPR | -.06 | -.33 | .20 | -.04 | -.45 | .651 |
| Gender | .38 | .13 | .63 | .28 | 2.94 | .003 |
| Moderate physical punishment x Gender | .10 | -.15 | .34 | .12 | .76 | .446 |
| 5-HTTLPR x Gender | -.11 | -.37 | .16 | -.13 | -.79 | .431 |
| BMI | .001 | -.04 | .05 | .003 | .03 | .974 |
| Moderate physical punishment x BMI | .01 | -.03 | .05 | .19 | .56 | .578 |
| 5-HTTLPR x BMI | .01 | -.04 | .06 | .17 | .45 | .655 |
| **Severe Parental Physical Punishment** | | | | | | |
| <u>Main effects model: Drive for Thinness</u> | | | | | | |
| Constant | -1.73 | -2.67 | -.78 | | -3.75 | .001 |
| Severe physical punishment | .30 | -.09 | .69 | .06 | 1.53 | .126 |
| 5-HTTLPR | -.11 | -.31 | .09 | -.04 | -1.05 | .293 |
| Gender | 1.18 | 1.00 | 1.37 | .48 | 12.46 | <.001 |
| BMI | .11 | .07 | .15 | .28 | 5.40 | <.001 |
| <u>Interaction effect model: Drive for Thinness</u> | | | | | | |
| Constant | -1.71 | -3.16 | -.27 | | -2.34 | .020 |
| Severe physical punishment | .42 | -2.53 | 3.37 | .08 | .28 | .780 |
| 5-HTTLPR | -.22 | -1.96 | 1.52 | -.08 | -.25 | .802 |
| Severe physical punishment x 5-HTTLPR | .07 | -.78 | .92 | .01 | .16 | .872 |
| Gender | 1.14 | .78 | 1.49 | .47 | 6.33 | <.001 |
| Severe physical punishment x Gender | .37 | -.45 | 1.18 | .12 | .88 | .379 |
| 5-HTTLPR x Gender | .03 | -.38 | .44 | .02 | .16 | .875 |
| BMI | .11 | .05 | .17 | .29 | 3.42 | .001 |
| Severe physical punishment x BMI | -.03 | -.15 | .08 | -.15 | -.60 | .551 |
| 5-HTTLPR x BMI | .003 | -.07 | .08 | .03 | .08 | .938 |
| <u>Main effects model: Bulimia</u> | | | | | | |
| Constant | 1.02 | .54 | 1.50 | | 4.16 | <.001 |
| Severe physical punishment | .41 | .16 | .65 | .14 | 3.19 | .001 |
| 5-HTTLPR | -.06 | -.18 | .07 | -.04 | -.89 | .376 |
| Gender | .34 | .22 | .46 | .25 | 5.60 | <.001 |
| BMI | .01 | -.01 | .03 | .06 | 1.25 | .212 |

| | | | | | | |
|---|---|---|---|---|---|---|
| <u>Interaction effect model: Bulimia</u> | | | | | | |
| Constant | 1.05 | .15 | 1.94 | | 2.30 | .022 |
| Severe physical punishment | 1.28 | -.55 | 3.10 | .45 | 1.38 | .169 |
| 5-HTTLPR | -.24 | -1.41 | .94 | -.16 | -.40 | .691 |
| Severe physical punishment x 5-HTTLPR | .77 | .23 | 1.30 | .22 | 2.82 | .0048 |
| Gender | .41 | .19 | .63 | .31 | 3.70 | <.001 |
| Severe physical punishment x Gender | -.08 | -.60 | .44 | -.05 | -.30 | .764 |
| 5-HTTLPR x Gender | -.10 | -.36 | .16 | -.12 | -.74 | .459 |
| BMI | .007 | -.03 | .05 | .04 | .38 | .706 |
| Severe physical punishment x BMI | -.06 | -.123 | .009 | -.45 | -1.70 | .091 |
| 5-HTTLPR x BMI | .01 | -.04 | .07 | .21 | .524 | .603 |

*Note.* Confidence intervals (CIs) are set at 95%
